# Impact of respirator versus surgical masks on SARS-CoV-2 acquisition in healthcare workers: a prospective multicentre cohort

**DOI:** 10.1186/s13756-022-01070-6

**Published:** 2022-02-05

**Authors:** Sabine Haller, Sabine Güsewell, Thomas Egger, Giulia Scanferla, Reto Thoma, Onicio B. Leal-Neto, Domenica Flury, Angela Brucher, Eva Lemmenmeier, J. Carsten Möller, Philip Rieder, Markus Rütti, Reto Stocker, Danielle Vuichard-Gysin, Benedikt Wiggli, Ulrike Besold, Stefan P. Kuster, Allison McGeer, Lorenz Risch, Matthias Schlegel, Andrée Friedl, Pietro Vernazza, Christian R. Kahlert, Philipp Kohler

**Affiliations:** 1 Division of Infectious Diseases and Hospital Epidemiology, Cantonal Hospital St. Gallen, St. Gallen, Switzerland; 2 Department of Public and Global Health, University of Zurich, Zurich, Switzerland; 3 Clinical Trials Unit, Cantonal Hospital of St. Gallen, St. Gallen, Switzerland; 4 Epitrack, Recife, Brazil; 5 Department of Economics, University of Zurich, Zurich, Switzerland; 6 Psychiatry Services of the Canton of St. Gallen (South), Pfäfers, Switzerland; 7 Private Clinic for Psychiatry and Psychotherapy, Clienia Littenheid AG, Littenheid, Switzerland; 8 Center for Neurological Rehabilitation, Zihlschlacht, Switzerland; 9 Hirslanden Clinic, Zurich, Switzerland; 10 Hospital Region Fürstenland Toggenburg, Wil, Switzerland; 11 Division of Infectious Diseases and Hospital Epidemiology, Thurgau Hospital Group, Münsterlingen, Switzerland; 12 Division of Infectious Diseases and Hospital Epidemiology, Cantonal Hospital Baden, Baden, Switzerland; 13 Geriatric Clinic St. Gallen, St. Gallen, Switzerland; 14 Division of Infectious Diseases and Hospital Epidemiology, University Hospital Zurich, Zurich, Switzerland; 15 Sinai Health System, Toronto, Canada; 16 Labormedizinisches Zentrum Dr Risch Ostschweiz AG, Buchs, Switzerland; 17 Private Universität Im Fürstentum Liechtenstein, Triesen, Liechtenstein; 18 Centre of Laboratory Medicine, University Institute of Clinical Chemistry, University of Bern, Bern, Switzerland; 19 Department of Infectious Diseases and Hospital Epidemiology, Children’s Hospital of Eastern Switzerland, St. Gallen, Switzerland; 20 Swiss National Centre for Infection Prevention (Swissnoso), Bern, Switzerland

**Keywords:** COVID-19, Healthcare workers, Respirator, Surgical mask, Aerosol

## Abstract

**Background:**

There is insufficient evidence regarding the role of respirators in the prevention of SARS-CoV-2 infection. We analysed the impact of filtering facepiece class 2 (FFP2) versus surgical masks on the risk of SARS-CoV-2 acquisition among Swiss healthcare workers (HCW).

**Methods:**

Our prospective multicentre cohort enrolled HCW from June to August 2020. Participants were asked about COVID-19 risk exposures/behaviours, including preferentially worn mask type when caring for COVID-19 patients outside of aerosol-generating procedures. The impact of FFP2 on (1) self-reported SARS-CoV-2-positive nasopharyngeal PCR/rapid antigen tests captured during weekly surveys, and (2) SARS-CoV-2 seroconversion between baseline and January/February 2021 was assessed.

**Results:**

We enrolled 3259 participants from nine healthcare institutions, whereof 716 (22%) preferentially used FFP2. Among these, 81/716 (11%) reported a SARS-CoV-2-positive swab, compared to 352/2543 (14%) surgical mask users; seroconversion was documented in 85/656 (13%) FFP2 and 426/2255 (19%) surgical mask users. Adjusted for baseline characteristics, COVID-19 exposure, and risk behaviour, FFP2 use was non-significantly associated with decreased risk for SARS-CoV-2-positive swab (adjusted hazard ratio [aHR] 0.8, 95% CI 0.6–1.0) and seroconversion (adjusted odds ratio [aOR] 0.7, 95% CI 0.5–1.0); household exposure was the strongest risk factor (aHR 10.1, 95% CI 7.5–13.5; aOR 5.0, 95% CI 3.9–6.5). In subgroup analysis, FFP2 use was clearly protective among those with frequent (> 20 patients) COVID-19 exposure (aHR 0.7 for positive swab, 95% CI 0.5–0.8; aOR 0.6 for seroconversion, 95% CI 0.4–1.0).

**Conclusions:**

Respirators compared to surgical masks may convey additional protection from SARS-CoV-2 for HCW with frequent exposure to COVID-19 patients.

**Supplementary Information:**

The online version contains supplementary material available at 10.1186/s13756-022-01070-6.

## Background

The transmission of the severe acute respiratory syndrome coronavirus type 2 (SARS-CoV-2) via respiratory droplets and, probably less important, via fomites is undisputed [1, 2]. The role of aerosols has been extensively debated [3–6]. Reports from healthcare and non-healthcare settings suggest that SARS-CoV-2 may indeed be transmitted via aerosols, particularly in poorly ventilated indoor environments, even in the absence of so-called aerosol-generating procedures (AGP) [7, 8].

Healthcare workers (HCW) have a high risk of exposure to and infection with SARS-CoV-2 [9]. For HCW involved in AGP, international guidelines unanimously recommend the use of so-called respirators, which include filtering facepiece class 2 (FFP2), N95, or KN95, with the ability to filter microparticles. As a consequence of the conflicting opinions about aerosol transmission, guidelines differ regarding recommendations for the use of respirators outside of AGP. Whereas the Centers for Disease Control and Prevention (CDC) and the European Centre for Disease Prevention and Control (ECDC) recommend respirators if available, the World Health Organization (WHO) and the Swiss National Centre for Infection Prevention (Swissnoso) recommend surgical masks [10–13]. The Infectious Diseases Society of America’s recommendation is to use either a surgical mask or a respirator [14].

Prospective head-to-head comparisons evaluating the protective effect of these mask types against SARS-CoV-2 acquisition are sparse. In a meta-analysis including mostly studies on non-SARS-CoV-2 coronaviruses, use of respirators was associated with a protective effect, although no study directly compared respirators to surgical masks [15]. A meta-analysis comparing the clinical effectiveness of respirators to surgical masks for other respiratory viruses, including coronaviruses, found no significant difference concerning infection risk in HCW [16].

For SARS-CoV-2, we identified three studies not covered in the above-mentioned meta-analysis. An online survey among HCW from multiple countries showed a protective effect of respirators compared to surgical masks for those performing AGP on patients with Coronavirus Disease 2019 (COVID-19) [17]. A cross-sectional study from the US found respirator use to be associated with decreased seropositivity rate, although no multivariable analysis was performed [18]. In a prospective single-centre HCW cohort, respirators were protective regarding SARS-CoV-2 seroconversion, although use of other personal protective equipment (PPE) was not documented and residual confounding was suspected [19].

To summarize, there is currently insufficient evidence to determine if the use of FFP2 respirators reduces the risk of SARS-CoV-2 infections. In this analysis of prospective cohort data from Swiss HCW, we sought to assess the effectiveness of FFP2 compared to surgical masks regarding SARS-CoV-2 protection for HCW involved in patient care.

## Methods

### Study design, participants and setting

**Fig. 1:**
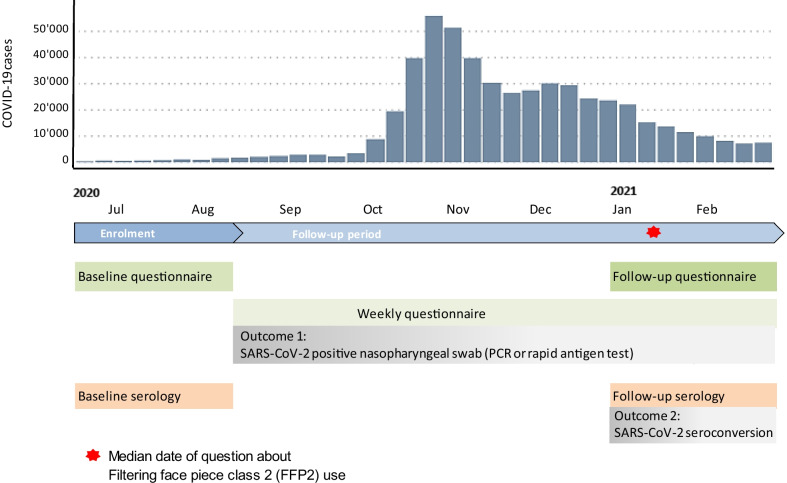
Study timeline and procedures in relation to laboratory confirmed (by polymerase chain reaction [PCR] or rapid antigen test) COVID-19 cases in Switzerland (absolute number of weekly cases)

We performed a prospective observational multicentre cohort consisting of employees (aged 16 years or older) from healthcare institutions in four different cantons in Northern and Eastern Switzerland. In the current analysis we only included HCW with patient contact. Employees registered online and provided electronic consent. Enrolment took place from June 22nd to August 15th 2020, between the end of the first COVID-19 wave in Switzerland and the surge of the second wave [20]; data were analysed up to March 9th 2021, when the second wave had abated (Fig. 1). During the study period, social distancing recommendations, as well as isolation and quarantine measures were continuously in place. In July 2020, the wearing of face masks became compulsory on public transport. In October 2020, mask wearing became compulsory in most indoor spaces and gatherings were restricted. From late December 2020, restaurants, as well as recreational und entertainment businesses were closed. From March 2021 onwards, restrictions were slowly eased.

### National and local mask policies

During the study period, a national policy required Swiss residents (including HCW) to wear at least a surgical mask at work. The Swiss National Centre for Infection Prevention (Swissnoso) suggested the use of a respirator mask only while performing AGP on confirmed or suspected COVID-19 patients [13]. However, this was considered minimum standard and institutions were free to recommend respirators outside of AGP. Also, in most institutions HCW could make the personal choice of wearing a respirator or surgical mask at work. To characterize institution-level mask recommendations, we conducted a survey among representatives from participating institutions asking about local policies for FFP2 use and estimated compliance with those policies (Additional file 1: Table S1). In accordance with national policy, surgical masks were required to meet the standard EN 14683 (usually type IIR, alternatively type II), FFP2 respirators the standard EN 149.

### Study procedures and questionnaires

The study timeline is shown in Fig. 1. Upon inclusion, participants answered a baseline questionnaire asking about anthropometric data, pregnancy and comorbidities, job description (including full-time equivalent percentage, profession, involvement in AGP, working in intensive care, exposure to COVID-19 patients, use of PPE, and visit to staff restaurant), and non-work related risk behaviour, e.g.wearing a mask outside of work, leisure and shopping activities, but also opinions on adequacy of regulatory measures. During follow-up, participants received weekly text messages and emails with a link to a questionnaire where they indicated results of nasopharyngeal swabs (polymerase chain reaction or laboratory confirmed rapid antigen tests) for SARS-CoV-2.

Additionally, participants answered whether they had been exposed to confirmed COVID-19 patients, co-workers, household contacts, or other COVID-19 cases during the previous week. In January 2021 (i.e. before follow-up serology was performed), a follow-up questionnaire asked about use of mask type (FFP2 vs. surgical mask) *outside* of AGP during COVID-19 patient contact, considering the entire period since beginning of the pandemic. AGP were defined as bronchoscopies, in-/extubation, gastroscopy, transesophageal echocardiography, reanimation, non-invasive ventilation, and suction of tracheal secretions. Participants had the choice among “Use of surgical mask only”; “Mostly use of surgical mask”; “Equal use of both mask types”; “Mostly use of FFP2”; “Use of FFP2 only”. For the purpose of this analysis, the two latter categories were classified as “Mostly FFP2”, and the first three categories merged to “Not mostly FFP2” (for better comprehensibility termed “Mostly surgical masks”). For HCW involved in AGP, we also asked whether they always used FFP2 during AGP, irrespective of the patient’s COVID-19 status (i.e. “universal FFP2 use”). Furthermore, use of other PPE including gowns, gloves and goggles while caring for COVID-19 patients was asked, as well as the number of COVID-19 patients HCW had been knowingly exposed to since March 2020 (Additional file 1: Table S2).

### Outcome assessment

Two main outcomes were defined: (1) time to first self-reported SARS-CoV-2 positive nasopharyngeal swab and (2) SARS-CoV-2 seroconversion. Results of nasopharyngeal swabs were asked in the weekly questionnaires. To verify that self-reported test results were accurate and complete, we cross-checked all reported positive tests and a random sample of negative test results with the database of the division of occupational health for a subgroup of HCW from the largest participating institution. Baseline (June–August 2020) and follow-up (January-February 2021) serologies were performed to assess SARS-CoV-2 seroconversion (Fig. 1). Participants with positive serology at baseline were excluded from this analysis. Samples were analysed with an electro-chemiluminescence immunoassay (ECLIA, Roche Diagnostics, Rotkreuz, Switzerland, antibodies against nucleocapsid-(N)-protein of SARS-CoV-2), as described elsewhere [20].

### Statistical analysis

For analysis of nasopharyngeal swabs, we performed Cox regression with time intervals between consecutive weekly questionnaires as response. Intervals were censored as long as no positive swab (event) was reported, those following the first event were excluded. The model included COVID-19 exposures reported in any of the three weekly questionnaires submitted before the end of each time interval as well as the cumulative number of negative swabs up to an interval's end (to account for different testing behaviour) as time-dependent co-variables, and answers from the baseline and follow-up questionnaire (including mask type) as time-independent co-variables. These variables were a priori chosen from the baseline questionnaire, based on their expected potential to confound the association between mask use and risk for SARS-CoV-2 acquisition [20]. Cantons and institutions were included as cluster terms.

For seroconversion, we used logistic regression including the same time-independent co-variables as for nasopharyngeal swabs as fixed effects and cluster terms as random effects. Instead of the time-dependent co-variables, we included overall household exposure (summarizing weekly reports into “any” vs. “none”) as well as the total number of COVID-19 patient and co-worker exposures. Variance inflation factors (VIF) were calculated to check that no co-variable had a VIF > 5. R statistical software Version 4.0.2 was used for all statistical analyses.

### Further analyses

We performed three sensitivity analyses to assess the influence of possible confounders on the estimated effect of mask type on the two outcomes: (1) including cantons and institutions as fixed effects (to account for regional incidence and institutional factors), (2) excluding HCW tested positively before December 1st 2020 (because mask use may have changed over time and was asked only in January 2021), and (3) excluding persons with a positively tested household member (since household transmissions contributed to a large part of infections while most likely not being affected by in-hospital mask use). Furthermore, we performed a complete case analysis for the two main outcomes, excluding all observations with missing values.We also performed a subgroup analysis according to frequency of COVID-19 patient contact (no known contact vs. 1–20 patients vs. > 20 patients since March 2020). Also, we repeated the analysis for those performing AGP, using a model including whether they always used FFP2 during AGP.

## Results

### Institutions

We included participants from seven acute care institutions (with 14 different sites), one rehabiliation clinic, and three psychiatry clinics (analysed as one institution). Institutional policies on respiratory protection are summarized in Additional file 1: Table S1. Most institutions followed the Swissnoso recommendations for use of FFP2. Actual FFP2 use varied considerably between institutions and ranged from 3 to 52% of participants. All acute care institutions with less strict local guidelines (i.e. no FFP2 required during contact with COVID-19 patients) reported that FFP2 were more frequently used than recommended (Additional file 1: Table S1).

### Baseline characteristics

Among the 3259 participants, 614 (19%) were male and median age was 39 years (interquartile range [IQR] 30–49 years). Most were nurses (n = 1724, 53%), followed by physicians (n = 671, 21%). Preferential use of FFP2 while caring for COVID-19 patients was reported by 716 (22%). HCW who preferentially used FFP2 were more likely to be male (OR 1.5, *p* < 0.001), to be ≤ 50 years old (OR 1.4, *p* = 0.001), to support stronger public restrictions regarding the pandemic (OR 1.7, *p* < 0.001), to be involved in AGP (OR 4.2, *p* < 0.001), to work in intensive care (OR 8.4, *p* < 0.001), to be exposed to > 20 COVID-19 patients (OR 2.8, *p* < 0.001), to use gowns (OR 8.7, *p* < 0.001), gloves (OR 2.5, *p* < 0.001) and goggles (OR 8.0, *p* < 0.001) while caring for COVID-19 patients, and to undergo testing for SARS-CoV-2 (OR 1.4, *p* < 0.001) (Table 1).

**Table 1:** Factors associated with preferential use of FFP2 vs. surgical masks among 3259 healthcare workers

Risk or protection factor for SARS-CoV-2 infection	Number of HCW without and with the factor		Frequency of the factor (n and %) in relation to FFP2 use during contact with COVID-19 patients		OR (95% CI)	*p* value (Fisher's test)
	Without	With	Mostly surgical mask (n = 2543)	Mostly FFP2 (n = 716)		
*Sociodemographic data*						
Age > 50 years	2528	731	604 (24%)	127 (18%)	0.69 (0.56–0.86)	0.001
Sex: male	2645	614	444 (17%)	170 (24%)	1.47 (1.20–1.81)	< 0.001
Living in Germany or Austria	3094	165	104 (4%)	61 (9%)	2.18 (1.55–3.06)	< 0.001
Child in household	2424	835	650 (26%)	185 (26%)	1.01 (0.83–1.23)	0.884
*Medical conditions*						
Comorbidity	2066	1193	921 (36%)	272 (38%)	1.08 (0.91–1.28)	0.404
Active smoker	2719	540	426 (17%)	114 (16%)	0.94 (0.74–1.18)	0.649
Pregnant during study	3122	137	113 (4%)	24 (3%)	0.75 (0.46–1.18)	0.246
*Behaviour outside of work*						
Prophylactic home remedies	2787	472	363 (14%)	109 (15%)	1.08 (0.85–1.37)	0.548
Social leisure activities	1693	1566	1208 (48%)	358 (50%)	1.11 (0.93–1.31)	0.253
Wearing a mask outside work	2354	905	657 (26%)	248 (35%)	1.52 (1.27–1.82)	< 0.001
Support for stronger public restrictions	2643	616	433 (17%)	183 (26%)	1.67 (1.37–2.05)	< 0.001
*HCW specifics*						
Job: Nurse	1535	1724	1309 (51%)	415 (58%)	1.30 (1.10–1.54)	0.002
Job: Physician	2588	671	500 (20%)	171 (24%)	1.28 (1.05–1.57)	0.016
Full-time job (> 80%)	1488	1771	1325 (52%)	446 (62%)	1.52 (1.28–1.81)	< 0.001
Involved in AGP	2055	1204	747 (29%)	457 (64%)	4.24 (3.55–5.07)	< 0.001
Work in intensive care	2971	288	102 (4%)	186 (26%)	8.39 (6.43–10.99)	< 0.001
Contact to > 20 COVID-19 patients	1812	1120	732 (32%)	388 (58%)	2.83 (2.36–3.39)	< 0.001
*Behaviour at work*						
Hygiene knowledge	491	2768	2121 (83%)	647 (90%)	1.87 (1.42–2.48)	< 0.001
Regular meals in staff restaurant	1094	2165	1702 (67%)	463 (65%)	0.90 (0.76–1.08)	0.263
Handwashing more frequent	343	2916	2268 (89%)	648 (91%)	1.16 (0.87–1.55)	0.335
*Test results*						
Positive SARS-CoV-2 test^a^	2826	433	352 (14%)	81 (11%)	0.79 (0.61–1.03)	0.081
Negative SARS-CoV-2 test^a^	1633	1626	1231 (48%)	395 (55%)	1.31 (1.11–1.55)	< 0.001
*Use of PPE*						
Always used goggles^b^	2362	897	446 (18%)	451 (63%)	7.99 (6.64–9.65)	< 0.001
Always used gloves^b^	1939	1320	904 (36%)	416 (58%)	2.51 (2.12–2.99)	< 0.001
Always used a gown^b^	2362	897	437 (17%)	460 (64%)	8.65 (7.17–10.46)	< 0.001
*Always used FFP2 during AGP*	645	559	242 (32%)	317 (69%)	4.72 (3.65–6.12)	< 0.001

*FFP2* filtering facepiece class 2, *HCW* health care worker, *AGP* aerosol-generating procedure, *PPE* personal protective equipment, *NA* not applicable

^a^At least one positive/negative test. The mean number of negative tests per HCW was 0.90 for surgical mask users and 1.11 for FFP2 users

^b^In contact with COVID-19 patients outside of AGP

### Risk of positive SARS-CoV-2 test according to mask type

**Fig. 2:**
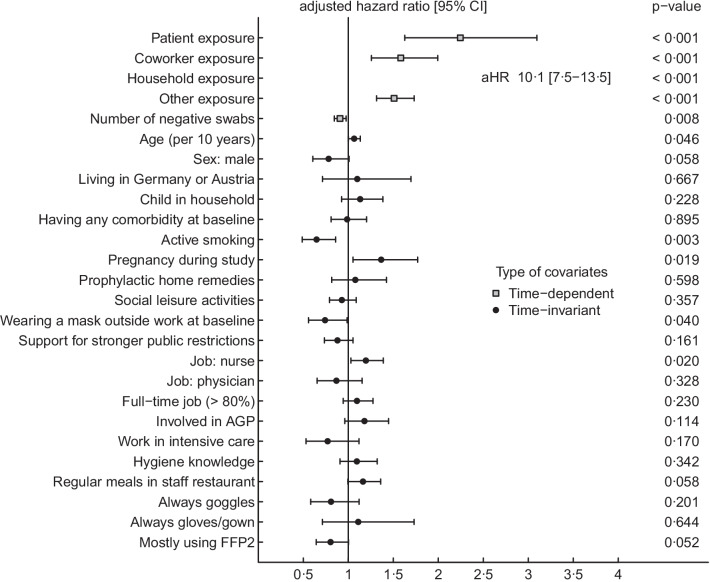
Forest plots showing results of the multivariable Cox regression analysis with outcome “SARS-CoV-2-positive nasopharyngeal PCR/rapid antigen test” (participants n = 3259, positive swabs n = 433) (*AGP* aerosol-generating procedure, *FFP2* filtering facepiece class 2)

Median follow-up was 242 days, both for respirator and for surgical mask users (Wilcoxon test, *p* = 0.49). The number of self-reported positive SARS-CoV-2 tests was 81/716 (11%) for FFP2 users compared to 352/2543 (14%) in users of surgical masks (hazard ratio [HR] 0.8; 95% confidence interval [CI] 0.6–1.0; *p* = 0.06 log-rank test). For validation purposes, self-reported test results were cross-checked for a subgroup of participants. The database of the division of occupational medicine provided documentation for 150 out of 174 reported positive swab results. The remainder of positive tests were most likely done outside of the work place. Of a randomly selected 175 HCWs reporting negative test results only, none was registered as positive in the database. In the Cox regression model, the factor most strongly associated with a positive SARS-CoV-2 test was exposure to a positive household contact (adjusted HR [aHR] 10.1, 95% CI 7.5–13.5, *p* < 0.001). Use of FFP2 while caring for COVID-19 patients was associated with a decreased risk of acquiring SARS-CoV-2 (aHR 0.8, 95% CI 0.7–1.0, *p* = 0.052) (Fig. 2, Additional file 1: Table S3). In sensitivity analyses, restriction to data collected after December 1st (aHR 0.7, *p* = 0.03) showed similar results, treating institutions/cantons as fixed effect (aHR 0.9, *p* = 0.43) resulted in a non-significant association. Excluding persons with a positively tested household member resulted in a similar point estimate with non-significant association (aHR 0.8, *p* = 0.36) (Additional file 1: Table S3). The result of the complete case analysis excluding observations with missing values was again significant (aHR 0.7, *p* = 0.009) (Additional file 1: Table S4).

### Risk of SARS-CoV-2 seroconversion according to mask type

**Fig. 3:**
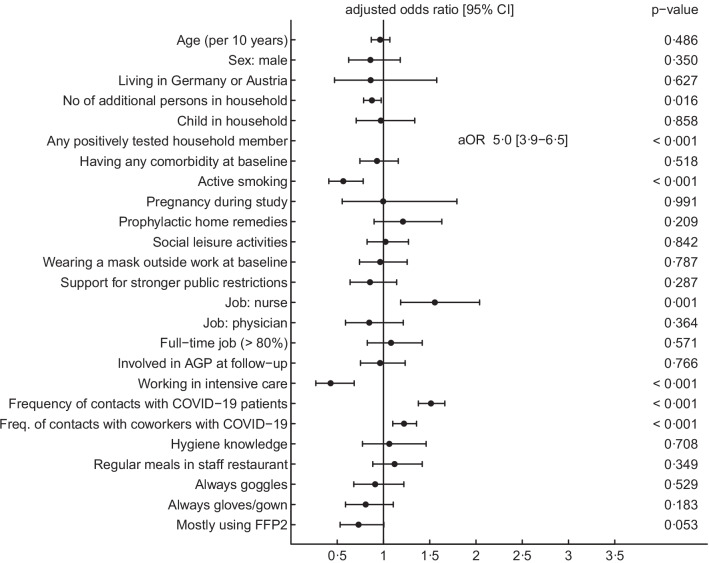
Forest plots showing results of the generalized mixed-effects model regarding outcome “SARS-CoV-2 seroconversion” (participants n = 2916, seroconversions n = 511) (*AGP* aerosol-generating procedure, *FFP2* filtering facepiece class 2)

We included 2916 HCW with negative baseline serology, who had a second serology performed in January/February 2021. Seroprevalence was 12.9% (85/658) for FFP2 users compared to 18.9% (429/2258) for users of surgical masks (OR 0.6, 95% CI 0.5–0.8, *p* < 0.001). In multivariable analysis, the strongest risk factor for seroconversion was again having a positive household member with an adjusted odds ratio (aOR) of 5.0 (95% CI 3.9–6.5, *p* < 0.001). FFP2 use was non-significantly associated with decreased risk for seroconversion (0.7, 95% CI 0.5–1.0, *p* = 0.053) (Fig. 3, Additional file 1: Table S5). In sensitivity analyses, including cantons/institutions as a fixed effect (aOR 0.8, *p* = 0.088) did not significantly change the point estimate for FFP2 use nor the significance level. Excluding persons with a positively tested household member (aOR 0.7, *p* = 0.46) (Additional file 1: Table S5) and complete case analysis both showed very similar results (aOR 0.7, *p* = 0.049) (Additional file 1: Table S4).

### Subgroup analyses

**Fig. 4:**
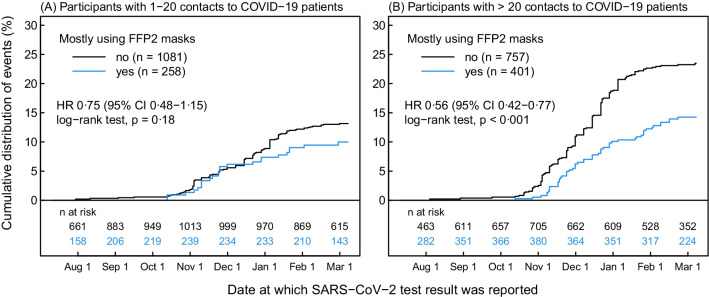
Kaplan–Meier curve regarding unadjusted risk of a SARS-CoV-2-positive nasopharyngeal swab for healthcare workers (HCW) with infrequent (**a**) and frequent (**b**) exposure to COVID-19 patients (*FFP2* filtering facepiece class 2)

HCW with frequent (> 20) exposure to COVID-19 patients were less likely to report a positive SARS-CoV-2 swab when mostly wearing FFP2, with an unadjusted HR of 0.6 (*p* < 0.001) compared to 0.8 (*p* = 0.18) for those with 1–20 patient contacts (Fig. 4). In multivariable analysis, the risk for a positive swab (aHR 0.7, *p* < 0.001) and for seroconversion (aOR 0.6, *p* = 0.036) remained significant for HCW with frequent exposure, which was not the case for those with less frequent exposure (aHR 1.1, *p* = 0.77 and aOR 0.8, *p* = 0.32) (Additional file 1: Table S6). For the group of HCW without known COVID-19 patient contact, the number of positive swabs was too small to perform multivariable analyses (2 events among 40 FFP2 users, 29 events among 480 surgical mask users).

For HCW performing AGP, universal use of FFP2 during AGP (irrespective of the patients COVID-19 status) showed no effect (aHR 1.1, *p* = 0.66 and aOR 0.9, *p* = 0.54, respectively) (Additional file 1: Table S7).

## Discussion

In this prospective multicentre cohort of unvaccinated HCW, FFP2 respirator use outside of AGP was marginally not associated with a decreased risk for SARS-CoV-2 infection compared to surgical masks. However, subgroup analysis suggested a protective effect for those with frequent COVID-19 patient exposure. Using FFP2 irrespective of the patient’s COVID-19 status did not provide additional protection for HCW involved in AGP. The large sample size, the dual approach for outcome assessment, and consideration of a variety of potential confounder variables (including personal risk factors, use of other PPE, and general risk perception) are among the strengths of this study.

This is, to our knowledge, the first prospective multicentre study comparing the effect of respirators and surgical masks regarding protection from SARS-CoV-2.

The overall association between FFP2 use and risk for SARS-CoV-2 infection was marginally not significant. This is probably a reflection of the heterogeneous study population, two thirds of which consisted of HCW with only sporadic (or even no known) COVID-19 exposure. However, for HCW with frequent exposure, we found a significant protective effect associated with FFP2 use. Several reports suggest that aerosol transmission is indeed a non-negligible mode of SARS-CoV-2 transmission and that respirators may provide additional protection compared to surgical masks [17–19, 21]. On the other hand, case reports have suggested that surgical masks are equivalent to respirators in protecting HCW from SARS-CoV-2 infection [22, 23]. These supposedly contradictory findings can be reconciled when considering a particular feature of SARS-CoV-2, namely its high overdispersion [24]. Overdispersion describes the highly variable transmissibility of infected individuals; in other words, only a minority of infected individuals actually transmit the virus to others, often within so-called superspreading events. As a consequence, the probability of being exposed but not infected is relatively high (irrespective of mask type). Supporting this hypothesis, a simulation study by Chen et al. describes the large variability in SARS-CoV-2 viral loads of infectious individuals and how this influences infection probability and the effectiveness of the different mask types [25].

Our effect size (aOR 0.7) was in the range of those reported by Lentz et al. (aOR of 0.4) and Martischang et al. (aOR of 0.7) [17, 19]. Since many HCW in our study did not consistently wear either FFP2 or surgical masks, we have to assume that the protective effect of FFP2 might be even higher in reality. However, the clinical significance of the protective effect mediated by FFP2 use can be questioned, given the dominating impact of extra-occupational exposures on the COVID-19 risk in HCW, as seen in other studies [26]. Moreover, the disadvantages of respirators (the discomfort over long periods of time, the possibility of a diminished protective effect without prior training and fit testing, and their cost) have to be considered when assessing the net benefit of FFP2 over surgical masks [27, 28].

Notably, we did not observe any protective effect of FFP2 for HCW performing AGP in the absence of COVID-19 suspicion in the patient. We extrapolate from these findings that universal FFP2 use in the hospital setting, where the average exposure risk is usually lower than during AGP, does not provide additional protection compared to surgical masks. We acknowledge however that in settings with a high proportion of undiagnosed, asymptomatic or presymptomatic patients, an additional benefit through universal FFP2 use cannot be excluded.

In sensitivity analysis, treating cantons and institutions as fixed effects alluded to a diminished association of the protective effect of FFP2 use (but only for the outcome of self-reported SARS-CoV-2 swab and not for seroconversion). This could be explained by differences in testing of HCW between institutions. However, the occurrence of regional differences and institutional factors contributing to the observed effect cannot be excluded. Similarly, excluding participants with positive household contact resulted in a non-significant association for the outcome of self-reported positive swabs. Yet again, the consistent result for seroconversion (which is a more objective outcome than self-reported swabs) strengthens the validity of our data.

To adjust for potential confounding, we included the use of gloves, gowns and goggles in our multivariable analysis. None of these measures were associated with any clear additional protective effect. Other associations with SARS-CoV-2 infection found in our study, such as the “protective” effect of active smoking or the increased risk associated with working as a nurse, have been discussed earlier [20].

Our study has limitations. First, residual confounding is possible. Yet, we have included multiple co-variables accounting for risk exposures and risk behaviours within and outside the hospital. Also, the fact that use of other PPE or universal respirator use among HCW performing AGP (representing HCW with particularly risk-averse behaviour) were not associated with reduced seroconversion rate, supports our argument of a valid multivariable model with low risk of residual confounding. Second, information about respirator use was collected in January 2021, when most SARS-CoV-2 positive participants had already had their infection. A positive test result could have led to a change in preferred mask type (in either direction). However, restricting the analysis to the time period close to the follow-up questionnaire showed similar or even stronger associations compared to the full model. Third, recall bias concerning use of mask type cannot be completely ruled out, though the ongoing debate about mask type in health care institutions, especially in the beginning of the study period, made their use a conscious choice and therefore likely to be remembered. Fourth, we did not specifically ask about type and duration of contact to individual COVID-19 patients, although type of profession, work percentage, or involvement in AGP can be regarded as proxy for this potentially important variable. Fifth, although we included multiple institutions, settings, and geographical regions in our study, the generalizability of the results can be questioned due to the fact that study participation was non-mandatory. However, distribution of key variables (e.g. age, sex, profession) were similar between the total HCW population (from the largest participating institution) and the cohort population [20]. Also, these results might not be valid for a vaccinated HCW population, as only data from non-vaccinated HCW were analysed. As more HCW become immunized the protective effect of FFP2 can be expected to diminish. Sixth, this study was performed before the emergence of the SARS-CoV-2 Delta and Omicron variants. The new variants show an increased transmission potential, which might lead to underestimation of the protective effect of FFP2 [29].

## Conclusions

FFP2 respirator use outside of AGP may reduce the risk of SARS-CoV-2 acquisition for HCW with high COVID-19 patient exposure, while those with only sporadic or no known contact do not seem to benefit. The effect size should be interpreted in the context of the global COVID-19 risk for HCW, which is driven by exposure to positive household contacts. No significant protective effect was observed for those using FFP2 during AGP in the absence of clinical COVID-19 suspicion. Pending results of randomized controlled trials [30], our data offer support for healthcare institutions and policy makers in gauging the expected add-on value of respirators compared to surgical masks.

## Supplementary Information

**Additional file 1.** **Table S1.** List of participating health care institutions with local mask policies (FFP2 or surgical masks), self-reported global adherence, number of healthcare workers (HCW) participating in the study, self-reported FFP2 use in contact with COVID-19 patients, and SARS-CoV-2 seroprevalence among healthcare workers. **Table S2.** Description and categorization of variables used in analyses. **Table S3.** Multivariable Cox regression analysis (full model and sensitivity analyses) with outcome “Time to first SARS-CoV-2- positive nasopharyngeal PCR/rapid antigen test”. **Table S4.** Complete case analysis excluding observations with missing values: A) Cox regression (outcome time to first SARS-CoV-2 positive swab); B) multivariable logistic regression (outcome SARS-CoV-2 seroconversion). **Table S5.** Results of multivariable logistic regression analysis (full model and sensitivity analyses) regarding outcome “SARS-CoV-2 seroconversion”. **Table S6.** Subgroup analysis of HCW with frequent COVID-19 exposure vs. HCW with less frequent COVID-19 exposure; A) Cox regression (outcome SARS-CoV-2 positive swab); B) multivariable logistic regression (outcome SARS-CoV-2 seroconversion). **Table S7.** Subgroup analysis of HCW performing AGP: A) Cox regression (outcome SARS-CoV-2 positive swab); B) multivariable logistic regression (outcome SARS-CoV-2 seroconversion).

## Data Availability

The datasets used and/or analysed during the current study are not publicly available but are available from the corresponding author on reasonable request.

## Abbreviations

AGP: Aerosol-generating procedures
CDC: Centers for Disease Control and Prevention
CI: Confidence interval
COVID-19: Coronavirus disease 2019
COPD: Chronic obstructive pulmonary disease
ECDC: European Centre for Disease Prevention and Control
FFP2: Filtering facepiece class 2
HCW: Healthcare worker
(a) HR: (adjusted) Hazard ratio
IQR: Interquartile range
NA: Not applicable
(a) OR: (adjusted) Odds ratio
PCR: Polymerase chain reaction
PPE: Personal protective equipment
SARS-CoV-2: Severe acute respiratory syndrome coronavirus type 2
VIF: Variance inflation factor
WHO: World Health Organization

## References

[1] Wiersinga WJ, Rhodes A, Cheng AC, Peacock SJ, Prescott HC. Pathophysiology, transmission, diagnosis, and treatment of coronavirus disease 2019 (COVID-19): a review. JAMA. 2020 [cited 2020 Jul 13]; Available from: https://jamanetwork.com/journals/jama/fullarticle/2768391. 10.1001/jama.2020.12839 32648899

[2] Mondelli MU, Colaneri M, Seminari EM, Baldanti F, Bruno R (2021). Low risk of SARS-CoV-2 transmission by fomites in real-life conditions. Lancet Infect Dis.

[3] Greenhalgh T, Jimenez JL, Prather KA, Tufekci Z, Fisman D, Schooley R (2021). Ten scientific reasons in support of airborne transmission of SARS-CoV-2. Lancet Lond Engl.

[4] Morawska L, Milton DK (2020). It is time to address airborne transmission of coronavirus disease 2019 (COVID-19). Clin Infect Dis.

[5] Sommerstein R, Fux CA, Vuichard-Gysin D, Abbas M, Marschall J, Balmelli C, et al. Risk of SARS-CoV-2 transmission by aerosols, the rational use of masks, and protection of healthcare workers from COVID-19. Antimicrob Resist Infect Control. 2020 [cited 2020 Jul 14];9. Available from: https://www.ncbi.nlm.nih.gov/pmc/articles/PMC7336106/. 10.1186/s13756-020-00763-0 PMC7336106 32631450

[6] Conly J, Seto WH, Pittet D, Holmes A, Chu M, Hunter PR. Use of medical face masks versus particulate respirators as a component of personal protective equipment for health care workers in the context of the COVID-19 pandemic. Antimicrob Resist Infect Control. 2020 [cited 2021 Apr 16];9. Available from: https://www.ncbi.nlm.nih.gov/pmc/articles/PMC7406874/. 10.1186/s13756-020-00779-6 PMC7406874 32762735

[7] Lu J, Gu J, Li K, Xu C, Su W, Lai Z, et al. COVID-19 outbreak associated with air conditioning in restaurant, Guangzhou, China, 2020—July 2020. Emerg Infect Dis. J CDC. 2020;26(7). [cited 2021 May 3]; Available from: https://wwwnc.cdc.gov/eid/article/26/7/20-0764_article. 10.3201/eid2607.200764 PMC7323555 32240078

[8] Liu Y, Ning Z, Chen Y, Guo M, Liu Y, Gali NK (2020). Aerodynamic analysis of SARS-CoV-2 in two Wuhan hospitals. Nature.

[9] Iversen K, Bundgaard H, Hasselbalch RB, Kristensen JH, Nielsen PB, Pries-Heje M (2020). Risk of COVID-19 in health-care workers in Denmark: an observational cohort study. Lancet Infect Dis.

[10] Centers for Disease Control and Prevention CDC. Updated healthcare infection prevention and control recommendations in response to COVID-19 Vaccination. 2021 [cited 2021 May 14]. Available from: https://www.cdc.gov/coronavirus/2019-ncov/hcp/infection-control-after-vaccination.html.

[11] European Centre for Disease Prevention and Control ECDC. Infection prevention and control and preparedness for COVID-19 in healthcare settings, Sixth update—9 February 2021 [Internet]. 2021 [cited 2021 Apr 29]. Available from: https://www.ecdc.europa.eu/sites/default/files/documents/Infection-prevention-and-control-in-healthcare-settings-COVID-19_6th_update_9_Feb_2021.pdf.

[12] World Health Organization WHO. Mask use in the context of COVID-19, Interim guidance, 1 December 2020 [Internet]. 2020 [cited 2021 Apr 28]. Available from: https://apps.who.int/iris/rest/bitstreams/1319378/retrieve.

[13] Swissnoso, Swiss National Centre for Infection Prevention. Interims Vorsorgemassnahmen in Spitälern für einen hospitalisierten Patienten mit begründetem Verdacht oder mit einer bestätigten COVID-19 Infektion, Version 8.7, Swissnoso, 25. März 2021 [Internet]. 2021 [cited 2021 Apr 29]. Available from: https://www.swissnoso.ch/fileadmin/swissnoso/Dokumente/5_Forschung_und_Entwicklung/6_Aktuelle_Erreignisse/210325_Vorsorgemassnahmen_COVID-19_Spital_V8.7_DE.pdf.

[14] Lynch JB, Davitkov P, Anderson DJ, Bhimraj A, Cheng VC-C, Guzman-Cottrill J (2020). Infectious diseases society of america guidelines on infection prevention for healthcare personnel caring for patients with suspected or known coronavirus disease 2019. Clin Infect Dis.

[15] Chu DK, Akl EA, Duda S, Solo K, Yaacoub S, Schünemann HJ (2020). Physical distancing, face masks, and eye protection to prevent person-to-person transmission of SARS-CoV-2 and COVID-19: a systematic review and meta-analysis. Lancet.

[16] Bartoszko JJ, Farooqi MAM, Alhazzani W, Loeb M (2020). Medical masks vs N95 respirators for preventing COVID-19 in healthcare workers: a systematic review and meta-analysis of randomized trials. Influenza Other Respir Viruses.

[17] Lentz RJ, Colt H, Chen H, Cordovilla R, Popevic S, Tahura S (2021). Assessing coronavirus disease 2019 (COVID-19) transmission to healthcare personnel: the global ACT-HCP case-control study. Infect Control Hosp Epidemiol.

[18] Bryan AD, Tatem K, Diuguid-Gerber J, Cooke C, Romanoff A, Choudhury N (2021). Seroepidemiology among employees of New York city health and hospitals during the first wave of the SARS-CoV-2 epidemic. Occup Environ Health.

[19] Martischang R, Iten A, Arm I, Abbas M, Meyer B, Yerly S (2021). Severe acute respiratory coronavirus virus 2 (SARS-CoV-2) seroconversion and occupational exposure of employees at a Swiss university hospital: a large longitudinal cohort study. Infect Control Hosp Epidemiol.

[20] Kahlert CR, Persi R, Güsewell S, Egger T, Leal-Neto OB, Sumer J, et al. Non-occupational and occupational factors associated with specific SARSCoV-2 antibodies among hospital workers—a multicentre cross-sectional study. Clin Microbiol Infect. 2021;27:1336–44. 10.1016/j.cmi.2021.05.014 PMC8131187 34020033

[21] Nissen K, Krambrich J, Akaberi D, Hoffman T, Ling J, Lundkvist Å (2020). Long-distance airborne dispersal of SARS-CoV-2 in COVID-19 wards. Sci Rep.

[22] Ng K, Poon BH, Kiat Puar TH, Shan Quah JL, Loh WJ, Wong YJ (2020). COVID-19 and the risk to health care workers: a case report. Ann Intern Med.

[23] Periyasamy P, Ng BH, Ali UK, Rashid ZZ, Kori N (2021). Aerosolized SARS-CoV-2 transmission risk: Surgical or N95 masks?. Infect Control Hosp Epidemiol.

[24] Endo A, Abbott S, Kucharski AJ, Funk S, Centre for the Mathematical Modelling of Infectious Diseases COVID-19 Working Group (2020). Estimating the overdispersion in COVID-19 transmission using outbreak sizes outside China. Wellcome Open Res.

[25] Cheng Y, Ma N, Witt C, Rapp S, Wild PS, Andreae MO (2021). Face masks effectively limit the probability of SARS-CoV-2 transmission. Science.

[26] Steensels D, Oris E, Coninx L, Nuyens D, Delforge M-L, Vermeersch P (2020). Hospital-wide SARS-CoV-2 antibody screening in 3056 staff in a tertiary center in Belgium. JAMA.

[27] Regli A, Sommerfield A, von Ungern-Sternberg BS (2021). The role of fit testing N95/FFP2/FFP3 masks: a narrative review. Anaesthesia.

[28] Ofner-Agostini M, Gravel D, McDonald LC, Lem M, Sarwal S, McGeer A (2006). Cluster of cases of severe acute respiratory syndrome among Toronto healthcare workers after implementation of infection control precautions: a case series. Infect Control Hosp Epidemiol.

[29] Li B, Deng A, Li K, Hu Y, Li Z, Xiong Q (2021). Viral infection and transmission in a large, well-traced outbreak caused by the SARS-CoV-2 delta variant. MedRxiv.

[30] Medical Masks vs N95 Respirators for COVID-19 - Tabular View - ClinicalTrials.gov [Internet]. [cited 2021 Apr 30]. Available from: https://clinicaltrials.gov/ct2/show/record/NCT04296643

