# Supplementary material for: Impact of respirator versus surgical masks on SARS-CoV-2 acquisition in healthcare workers: a prospective multicentre cohort
Source: Antimicrob Resist Infect Control. 2022 Feb 5;11:27. doi: 10.1186/s13756-022-01070-6 (PMC8817591; doi:10.1186/s13756-022-01070-6)
Supplement: Supplementary file 1 — Additional file 1. Table S1. List of participating health care institutions with local mask policies (FFP2 or surgical masks), self-reported global adherence, number of healthcare workers (HCW) participating in the study, self-reported FFP2 use in contact with COVID-19 patients, and SARS-CoV-2 seroprevalence among healthcare workers. Table S2. Description and categorization of variables used in analyses. Table S3. Multivariable Cox regression analysis (full model and sensitivity analyses) with outcome “Time to first SARS-CoV-2- positive nasopharyngeal PCR/rapid antigen test”. Table S4. Complete case analysis excluding observations with missing values: A) Cox regression (outcome time to first SARS-CoV-2 positive swab); B) multivariable logistic regression (outcome SARS-CoV-2 seroconversion). Table S5. Results of multivariable logistic regression analysis (full model and sensitivity analyses) regarding outcome “SARS-CoV-2 seroconversion”. Table S6. Subgroup analysis of HCW with frequent COVID-19 exposure vs. HCW with less frequent COVID-19 exposure; A) Cox regression (outcome SARS-CoV-2 positive swab); B) multivariable logistic regression (outcome SARS-CoV-2 seroconversion). Table S7. Subgroup analysis of HCW performing AGP: A) Cox regression (outcome SARS-CoV-2 positive swab); B) multivariable logistic regression (outcome SARS-CoV-2 seroconversion). [file 13756_2022_1070_MOESM1_ESM.docx]

**Additional file 1**

Table S1. List of participating health care institutions with local mask policies (FFP2 or surgical masks), self-reported global adherence, number of healthcare workers (HCW) participating in the study, self-reported FFP2 use in contact with COVID-19 patients, and SARS-CoV-2 seroprevalence among healthcare workers.

| Institution | AGP | |  | Non-AGP | | Hospital  area | Fit test | Adherence^1^ | HCW  (n) | Reported  FFP2 use  (in %) | Sero- prevalence (in %) |
| --- | --- | --- | --- | --- | --- | --- | --- | --- | --- | --- | --- |
|  | COVID-19  patients | Other  patients |  | COVID-19  patients | Other  patients |  |  |  |  |  |  |
| A | FFP2 | Surgical |  | FFP2 | Surgical | Surgical | Yes | Good | 612 | 34.6 | 10.8 |
| B | FFP2 | Surgical |  | Surgical | Surgical | Surgical | No | More  FFP2 | 241 | 51.5 | 17.0 |
| C | FFP2 | Surgical |  | Surgical | Surgical | Surgical | No | More  FFP2 | 105 | 13.3 | 37.1 |
| D | FFP2 | Surgical |  | Surgical | Surgical | Surgical | No | More  FFP2 | 329 | 12.2 | 30.4 |
| E^2^ | FFP2 | Surgical |  | Surgical | Surgical | Surgical | No | More  FFP2 | 882 | 10.5 | 20.1 |
| F | FFP2 | Surgical |  | Surgical | Surgical | Surgical | No | More  FFP2 | 94 | 16.0 | 14.9 |
| G | FFP2 | FFP2 |  | FFP2 | Surgical | Surgical | No | Good | 299 | 43.8 | 11.7 |
| H^3^ | NA | NA |  | Surgical | Surgical | Surgical | No | Good | 262 | 9.2 | 11.1 |
| I^4^ | FFP2 | FFP2 |  | Surgical | Surgical | Surgical | No | Good | 87 | 3.4 | 11.5 |
| Swiss-noso^5^ | FFP2 | Surgical |  | Surgical | Surgical | .. | .. | .. | .. | .. | .. |

^1^ Choice of "good", "more FFP2 used than recommended", "less FFP2 used than recommended”

^2^ Includes geriatric hospital
^3^ Three psychiatry inpatient/outpatient clinics
^4^ Rehabiliation centre

^5^ National recommendations [13]

Abbreviations: FFP2 – Filtering facepiece class 2. AGP – Aerosol-generating procedure. HCW – Health care worker. Surgical – Surgical mask. NA – Not applicable.

Table S2. Description and categorization of variables used in analyses.

| **Variable** | **Question in Questionnaire** | **Answer possibilities** | **Calculation / Categorisation** | **Timepoint** |
| --- | --- | --- | --- | --- |
| **Test results** |  |  |  |  |
| Positive SARS-CoV-2 test | Did you have a positive SARS CoV-2 swab test? | True; False | True; False | Weekly |
| Number of negative swab tests | Have you been tested for SARS-CoV-2 in the past week? | True; False | Cumulative number of negative tests up to this date | Weekly |
|  | What was the result? | Positive; Negative; Pending |  | Weekly |
| **Exposure to COVID-19** |  |  |  |  |
| Frequency of contacts to COVID-19 patients | How many COVID-19 patients were you exposed to since March 2020? | None; 1 to 5; 6 to 10; 11 to 20; more than 20; I don’t know | None, 1-20, >20 | Follow-up |
| Frequency of contacts to coworkers with COVID-19 | How many SARS-CoV-2 confirmed coworkers were you exposed to since March 2020? | 1; 2; 3; 4 or more | 1;2;3;4 | Follow-up |
| Any positively tested household member | Has someone of your household or your intimate partner been tested positive since March 2020? | True; False | True; False | Weekly |
| Patient exposure *(time dependent)* | Did you have contact to a confirmed COVID-19 patient in hospital last week? | True; False | True; False | Weekly |
| Coworker exposure *(time dependent)* | Did you have contact to a SARS-CoV-2 confirmed coworker whithout wearing a mask last week? | True; False | True; False | Weekly |
| Household exposure *(time dependent)* | Has someone from your household been tested positive for SARS-CoV-2 last week? | True; False | True; False | Weekly |
| Other exposure *(time dependent)* | Has someone other from your surroundings been tested positive for SARS-CoV-2 last week? | True; False | True; False | Weekly |
| **Sociodemographic data** |  |  |  |  |
| Age | What is your date of birth? | Day/Month/Year | (2020−birthyear)/10 | Baseline |
| Sex | What is your sex? | Male; Female; Not specified | Male; Female and other | Baseline |
| Living in Germany or Austria | Zip code of your hometown | Numbers | GE/AU *vs.* CH | Baseline |
| Number of additional persons in household | How many people live in your place of residence? | Numbers | Numbers from 0 to 4 (larger numbers truncated to 4) | Baseline |
| Child in household | Year of birth for every household contact | Year | At least one year ≥ 2008 | Baseline |
| **Medical conditions** |  |  |  |  |
| Comorbidity | Do you suffer from any of the following diseases? | None; Arterial hypertension; Diabetes mellitus; Heart disease; COPD; Asthma; Liver disease (Fatty liver; Cirrhosis); Cancer; Rheumatologic disease (Arthritis, Lupus, Vasculitis); Hay fever; Hypothyroidism; Chronic inflamatory bowel disease (M. Crohn, Colitis ulcerosa), Intermittent claudication (Peripheral artery disease), Stroke | None = False; All others = True | Baseline |
| Active smoker | Do you smoke or did you use to smoke? | No; Used to smoke; Active smoker | Active smoker; other | Baseline |
| Pregnancy during study | Are you pregnant at the moment? | True; False | True; False | Follow-up |
| **Behaviour outside of work** |  |  |  |  |
| Prophylactic home remedies | Which preventive preparation do you take against COVID-19? | None; Echinacea; Zinc; Vitamin C; Umckaloabo/Kaloba; Vitamin B12; Homeopathics; Others | None = False; All others = True | Baseline |
| Social leisure activities | Which activities do you perform regularly at the moment? | None; Sportsclub; Choir; Orchestra/Music group (Active musician); Concert/Theatre; Cinema; Church; Yoga/Fitness; Restaurant/Bar | None = False; All others = True | Baseline |
| Wearing a mask outside work | In which situations did you wear a mask? | At home; Outside; On public transport; While shopping; In hospital area; In the workingspace; During contact with other people | None = False; All others = True | Baseline |
| Support for stronger public restrictions | Do you support the national measures and its duration or what is your opinion about it? | 1 Very exaggerated/much too long; 2 a bit exaggerated/a bit too long; 3 just perfect; 4 a bit weak/a bit too short; 5 much too weak/much too short | Answers 1-3 = False; Answers 4-5 = True | Baseline |
| **HCW specifics** |  |  |  |  |
| Job: nurse | What is your job description? | Nurse; Medical doctors assistant; Physician; Secretary; Scientist; Physiotherapist; Ergotherapist; Logopedist; Social service; Dietician; Medical technical radiology assistant (MTRA); Housekeeping; Volunteer; Gastronomy/Hotel services; Kitchen; Technical service; Administration (Human resources, management); Informatics; Laboratory; other | Nurse = True; All others = False | Baseline |
| Job: physician |  |  | Physician = True; All others = False | Baseline |
| Full-time job (> 80%) | What is your work percentage? | Percentage (xxx%) | >80% = True; 0-80% = False | Baseline |
| Involved in AGP | In which aerosol-generating procedures have you been involved since March 2020? | None; In-/Extubation; Bronchoscopy; Reanimation; Tracheal secretion suction; Non-invasive ventilation; Gastroscopy; Transesophageal echocardiography; other | None = False; All others = True | Follow-up |
| Working in intensive case | In which department do you work? | List of departments | Intensive care unit = True, All others = False | Baseline |
| **Behaviour at work** |  |  |  |  |
| Hygiene knowledge | Which are standard hygiene measures? | Cough etiquette; Physical distancing*; Handhygiene; Vaccines; Wearing a gown when in contact with body fluids; Wearing surgical mask when having a cold (* incorrect answer, all others are correct) | Number of correct answers ≥ 3 = True; < 3 = False | Baseline |
| Regular meals in staff restaurant | How often do you go to the staff restaurant or the cafeteria? | None is available; Never; Less than weekly; Weekly, but not daily; Daily; More than daily; No restaurant or cafeteria visits | None, never or less than weekly; weekly or more | Baseline |
| Handwashing more frequent | Do you wash your hands more frequently in the current COVID-19 situation compared to before? | True; False | True; False | Baseline |
| **Use of personal protective equipment** |  |  |  |  |
| Always used goggles^1^ | In what situations (during COVID-19 patient contact) did you use goggles? | 1 Never; 2 Occasionally; 3 Only if a contact with body fluids or secretions is expected; 4 Always | Answer 4 = True; Answers 1-3 = False | Follow-up |
| Always used gloves/gown^1^ | In what situations (during COVID-19 patient contact) did you use gloves?  In what situations (during COVID-19 patient contact) did you use a gown? | 1 Never; 2 Occasionally; 3 Only if a contact with body fluids or secretions is expected; 4 Always | Answer 4 (for both gloves and gown) = True; All others = False | Follow-up |
| Mostly used FFP2^1^ | What mask do you usually wear in contact with COVID-19 patients, when non-aerosol generating procedures are done? Please consider the whole time since the start of the pandemic. | 1 Always surgical mask; 2 mostly surgical mask; 3 Both surgical mask and FFP2; 4 Mostly FFP2; 5 Always FFP2 | Answers 1-3 = False; Answers 4-5 = True | Follow-up |
| Always used FFP2 during AGP | What mask do you usually wear in contact with COVID-19 patients, when aerosol generating procedures are performed? Please consider the whole time since the start of the pandemic. | 1 Always surgical mask; 2 Mostly surgical mask; 3 Both surgical mask andFFP2; 4 Mostly FFP2; 5 Always FFP2 | Answers 1-4 = False; Answer 5 = True | Follow-up |

^1^ In contact with COVID-19 patients outside of AGP

Abbreviations: AU – Austria. GE – Germany. HCW – Health care worker. COPD – Chronic obstructive pulmonary disease. AGP – Aerosol-generating procedure. FFP2 – Filtering facepiece class 2.

Table S3. Multivariable Cox regression analysis (full model and sensitivity analyses) with outcome “Time to first SARS-CoV-2- positive nasopharyngeal PCR/rapid antigen test”.

|  | **Full model**^1^ | | **Sensitivity analysis 1** | | **Sensitivity analysis 2**^1^ | | **Sensitivity analysis 3** | |
| --- | --- | --- | --- | --- | --- | --- | --- | --- |
|  |  |  | Treating cantons and  institutions as fixed effects | | Restricting analysis to events after Dec 1st 2020 | | persons without positively tested  household member | |
| Participants; Events | n=3’259, n=433 | | n=3’259; n=433 | | n=3’067; n=203 | | n=2’783; n=250 | |
| **Risk or protection factor** | **aHR (95% CI)** | **p** | **aHR (95% CI)** | **p** | **aHR (95% CI)** | **p** | **aHR (95% CI)** | **p** |
| **Exposure to COVID-19** |  |  |  |  |  |  |  |  |
| Patient exposure *(time dependent)* | 2.24 (1.63-3.10) | <0.001 | 2.11 (1.67-2.66) | <0.001 | 2.07 (1.55-2.76) | <0.001 | 2.84 (2.05-3.93) | <0.001 |
| Coworker exposure *(time dependent)* | 1.58 (1.26-1.99) | <0.001 | 1.56 (1.20-2.04) | 0.001 | 1.48 (1.13-1.94) | 0.004 | 1.98 (1.58-2.48) | <0.001 |
| Household exposure *(time dependent)* | 10.06 (7.48-13.51) | <0.001 | 9.64 (7.66-12.12) | <0.001 | 14.21 (9.66-20.91) | <0.001 |  |  |
| Other exposure *(time dependent)* | 1.51 (1.31-1.73) | <0.001 | 1.48 (1.20-1.83) | <0.001 | 1.64 (1.22-2.22) | 0.001 | 1.70 (1.45-2.00) | <0.001 |
| Number of negative swabs *(time dependent)* | 0.91 (0.84-0.98) | 0.008 | 0.91 (0.81-1.02) | 0.120 | 0.95 (0.87-1.04) | 0.243 | 0.94 (0.83-1.05) | 0.277 |
| **Sociodemographic data** |  |  |  |  |  |  |  |  |
| Age (per 10 years) | 1.06 (1.00-1.13) | 0.046 | 1.08 (0.98-1.18) | 0.133 | 0.96 (0.88-1.05) | 0.400 | 0.97 (0.89-1.05) | 0.429 |
| Gender: male | 0.78 (0.61-1.01) | 0.058 | 0.82 (0.60-1.11) | 0.191 | 0.64 (0.36-1.15) | 0.137 | 0.50 (0.38-0.66) | <0.001 |
| Living in Germany or Austria | 1.10 (0.71-1.70) | 0.667 | NA |  | 0.69 (0.35-1.38) | 0.295 | 0.92 (0.40-2.11) | 0.837 |
| Child in household | 1.13 (0.93-1.38) | 0.228 | 1.10 (0.86-1.40) | 0.458 | 1.70 (1.25-2.29) | 0.001 | 0.69 (0.55-0.87) | 0.002 |
| **Medical conditions** |  |  |  |  |  |  |  |  |
| Comorbidity | 0.99 (0.81-1.20) | 0.895 | 1.00 (0.82-1.23) | 0.982 | 0.99 (0.72-1.34) | 0.923 | 1.01 (0.76-1.33) | 0.965 |
| Active smoking | 0.65 (0.49-0.86) | 0.003 | 0.66 (0.50-0.89) | 0.006 | 0.55 (0.29-1.06) | 0.074 | 0.59 (0.41-0.85) | 0.005 |
| Pregnancy during study | 1.37 (1.05-1.77) | 0.019 | 1.37 (0.87-2.16) | 0.172 | 2.03 (1.33-3.09) | 0.001 | 1.27 (0.68-2.36) | 0.458 |
| **Behaviour outside of work** |  |  |  |  |  |  |  |  |
| Prophylactic home remedies | 1.08 (0.82-1.42) | 0.598 | 1.07 (0.82-1.40) | 0.599 | 1.30 (0.94-1.78) | 0.107 | 1.00 (0.76-1.30) | 0.974 |
| Social leisure activities | 0.93 (0.79-1.09) | 0.357 | 0.91 (0.74-1.10) | 0.324 | 1.04 (0.79-1.37) | 0.781 | 0.79 (0.67-0.94) | 0.007 |
| Wearing a mask outside work | 0.74 (0.56-0.99) | 0.040 | 0.75 (0.58-0.97) | 0.026 | 0.84 (0.64-1.11) | 0.222 | 0.69 (0.43-1.11) | 0.127 |
| Support for stronger public restrictions | 0.88 (0.73-1.05) | 0.161 | 0.96 (0.73-1.25) | 0.749 | 1.11 (0.76-1.62) | 0.593 | 0.92 (0.67-1.27) | 0.619 |
| **HCW specifics** |  |  |  |  |  |  |  |  |
| Job: nurse | 1.20 (1.03-1.39) | 0.020 | 1.15 (0.89-1.49) | 0.276 | 1.24 (0.94-1.63) | 0.127 | 1.43 (1.12-1.83) | 0.004 |
| Job: physician | 0.87 (0.65-1.15) | 0.328 | 0.82 (0.58-1.15) | 0.256 | 0.90 (0.55-1.46) | 0.669 | 1.10 (0.73-1.67) | 0.650 |
| Full-time job (> 80%) | 1.10 (0.94-1.27) | 0.230 | 1.08 (0.85-1.39) | 0.521 | 1.51 (1.05-2.16) | 0.026 | 1.10 (0.90-1.34) | 0.347 |
| Involved in AGP | 1.18 (0.96-1.45) | 0.114 | 1.13 (0.91-1.40) | 0.266 | 1.31 (1.04-1.67) | 0.024 | 1.21 (0.90-1.62) | 0.217 |
| Working in intensive care | 0.77 (0.53-1.12) | 0.170 | 0.74 (0.50-1.10) | 0.142 | 1.02 (0.63-1.66) | 0.924 | 0.63 (0.42-0.94) | 0.025 |
| **Behaviour at work** |  |  |  |  |  |  |  |  |
| Hygiene knowledge | 1.09 (0.91-1.32) | 0.342 | 1.00 (0.75-1.34) | 0.976 | 1.30 (0.96-1.75) | 0.089 | 1.01 (0.71-1.44) | 0.964 |
| Regular meals in staff restaurant | 1.16 (1.00-1.36) | 0.058 | 1.15 (0.93-1.43) | 0.199 | 1.16 (0.84-1.59) | 0.373 | 1.13 (0.95-1.34) | 0.173 |
| **Use of personal protective equipment** |  |  |  |  |  |  |  |  |
| Always used goggles^2^ | 0.81 (0.58-1.12) | 0.201 | 0.88 (0.67-1.16) | 0.375 | 0.66 (0.40-1.08) | 0.100 | 0.81 (0.53-1.25) | 0.342 |
| Always used gloves/gown^2^ | 1.11 (0.71-1.73) | 0.644 | 1.07 (0.81-1.41) | 0.636 | 1.16 (0.69-1.96) | 0.572 | 1.11 (0.69-1.80) | 0.664 |
| **Mostly used FFP2**^2^ | 0.80 (0.64-1.00) | 0.052 | 0.88 (0.66-1.18) | 0.402 | 0.73 (0.55-0.97) | 0.029 | **0.82 (0.54-1.25)** | **0.360** |

^1^ Institutions and living place (canton) included in model as random cluster term

^2^ Germany/Austria treated like separate cantons and included as fixed effects

^3^ In contact with COVID-19 patients outside of AGP

Abbreviations: PCR – Polymerase chain reaction. HCW – Health care worker. AGP – Aerosol-generating procedure. FFP2 – Filtering facepiece class 2. NA – Not applicable.

Table S4. Complete case analysis excluding observations with missing values: A) Cox regression (outcome time to first SARS-CoV-2 positive swab); B) multivariable logistic regression (outcome SARS-CoV-2 seroconversion).

| **A) Cox Regression** |  |  |  |
| --- | --- | --- | --- |
|  | **Complete case analysis** | | **Missing values** |
|  | n HCW = 2’063, n events = 233 | | n (%) |
| **Risk or protection factor** | **aHR (95% CI)** | **p** |  |
| **Exposure to COVID-19** |  |  |  |
| Patient exposure *(time dependent)* | 2.32 (1.62-3.32) | <0.001 | (3.5%) |
| Coworker exposure *(time dependent)* | 1.82 (1.19-2.78) | 0.006 | (4.2%) |
| Household exposure *(time dependent)* | 9.18 (6.88-12.23) | <0.001 | (6.2%) |
| Other exposure *(time dependent)* | 1.37 (1.15-1.63) | <0.001 | (9.5%) |
| Number of negative swabs *(time dependent)* | 0.78 (0.65-0.94) | 0.008 | (14.0%) |
| **Sociodemographic data** |  |  |  |
| Age (per 10 years) | 1.02 (0.93-1.13) | 0.645 | 0 (0.0%) |
| Sex: male | 0.84 (0.60-1.16) | 0.279 | 19 (0.6%) |
| Living in Germany or Austria | 0.99 (0.39-2.51) | 0.988 | 0 (0.0%) |
| Child in household | 1.20 (0.89-1.62) | 0.225 | 6 (0.2%) |
| **Medical conditions** |  |  |  |
| Comorbidity | 0.98 (0.70-1.39) | 0.928 | 141 (4.3%) |
| Active smoking | 0.76 (0.53-1.10) | 0.145 | 0 (0.0%) |
| Pregnancy during study | 1.63 (1.20-2.23) | 0.002 | 133 (4.1%) |
| **Behaviour outside of work** |  |  |  |
| Prophylactic home remedies | 0.85 (0.62-1.17) | 0.326 | 0 (0.0%) |
| Social leisure activities | 0.94 (0.68-1.29) | 0.69 | 107 (3.3%) |
| Wearing a mask outside work | 0.92 (0.68-1.26) | 0.618 | 14 (0.4%) |
| Support for stronger public restrictions | 0.83 (0.66-1.04) | 0.106 | 39 (1.2%) |
| **HCW specifics** |  |  |  |
| Job: nurse | 1.74 (1.01-2.99) | 0.046 | 2 (0.1%) |
| Job: physician | 1.09 (0.60-1.98) | 0.769 | 2 (0.1%) |
| Full-time job (> 80%) | 1.22 (0.94-1.57) | 0.135 | 0 (0.0%) |
| Involved in AGP | 1.26 (1.00-1.58) | 0.048 | 73 (2.2%) |
| Working in intensive care | 0.65 (0.37-1.15) | 0.14 | 721 (22.1%) |
| **Behaviour at work** |  |  |  |
| Hygiene knowledge | 1.22 (0.78-1.91) | 0.388 | 16 (0.5%) |
| Regular meals in staff restaurant | 1.40 (1.17-1.68) | <0.001 | 0 (0.0%) |
| **Use of personal protective equipment** |  |  |  |
| Always goggles | 0.75 (0.51-1.10) | 0.142 | 87 (2.7%) |
| Always gloves/gown | 1.40 (0.85-2.31) | 0.191 | 75 (2.3%) |
| **Mostly using FFP2** | **0.66 (0.49-0.90)** | **0.009** | 90 (2.8%) |
| Mostly using FFP2 during AGP |  |  | 7 (0.2%) |
|  |  |  |  |
| **B) Multivariable Logistic Regression** |  |  |  |
|  | **Complete case analysis** | | **Missing values** |
|  | n HCW = 1’392, n events = 284 | | n (%) |
| **Risk or protection factor** | **aOR (95% CI)** | **p-value** |  |
| **Exposure to COVID-19** |  |  |  |
| Frequency of contacts to COVID-19 patients | 1.67 (1.46 - 1.91) | <0.001 | 41 (1.4%) |
| Frequency of contacts to coworkers with COVID-19 | 1.15 (0.99 - 1.34) | 0.071 | 65 (2.2%) |
| Any positively tested household member | 4.05 (2.92 - 5.61) | <0.001 | 883 (30.3%) |
| **Sociodemographic data** |  |  |  |
| Age (per 10 years) | 1.02 (0.88 - 1.18) | 0.822 | 0 (0.0%) |
| Sex: male | 0.90 (0.58 - 1.37) | 0.612 | 18 (0.6%) |
| Living in Germany or Austria | 0.80 (0.33 - 1.94) | 0.623 | 0 (0.0%) |
| Number of additional persons in household | 0.92 (0.79 - 1.06) | 0.251 |  |
| Child in household | 0.93 (0.61 - 1.42) | 0.737 | 5 (0.2%) |
| **Medical conditions** |  |  |  |
| Comorbidity | 0.84 (0.62 - 1.14) | 0.267 | 122 (4.2%) |
| Active smoking | 0.55 (0.35 - 0.84) | 0.006 | 0 (0.0%) |
| Pregnancy during study | 1.15 (0.50 - 2.67) | 0.744 | 89 (3.1%) |
| **Behaviour outside of work** |  |  |  |
| Prophylactic home remedies | 0.83 (0.52 - 1.33) | 0.441 | 0 (0.0%) |
| Social leisure activities | 1.04 (0.77 - 1.41) | 0.784 | 98 (3.4%) |
| Wearing a mask outside work | 0.96 (0.66 - 1.39) | 0.812 | 10 (0.3%) |
| Support for stronger public restrictions | 0.69 (0.46 - 1.03) | 0.071 | 31 (1.1%) |
| **HCW specifics** |  |  |  |
| Job: nurse | 1.54 (0.64 - 3.70) | 0.339 | 2 (0.1%) |
| Job: physician | 0.91 (0.35 - 2.32) | 0.837 | 2 (0.1%) |
| Full-time job (> 80%) | 1.19 (0.81 - 1.75) | 0.378 | 0 (0.0%) |
| Involved in AGP | 0.97 (0.69 - 1.35) | 0.846 | 65 (2.2%) |
| Work in intensive care | 0.49 (0.28 - 0.84) | 0.010 | 651 (22.3%) |
| **Behaviour at work** |  |  |  |
| Hygiene knowledge | 0.99 (0.62 - 1.57) | 0.949 | 15 (0.5%) |
| Regular meals in staff restaurant | 1.30 (0.93 - 1.80) | 0.126 | 0 (0.0%) |
| **Use of personal protective equipment** |  |  |  |
| Always goggles^1^ | 0.83 (0.56 - 1.22) | 0.346 | 84 (2.9%) |
| Always gloves/gown^1^ | 0.85 (0.55 - 1.30) | 0.445 | 74 (2.5%) |
| **Mostly using FFP2**^1^ | **0.65 (0.42 - 1.00)** | **0.049** | **82 (2.8%)** |
| Mostly using FFP2 during AGP |  |  | 9 (0.3%) |

^1^ In contact with COVID-19 patients outside of AGP

Abbreviations: HCW – Health care worker. AGP – Aerosol-generating procedure. FFP2 – Filtering facepiece class 2.

Table S5. Results of multivariable logistic regression analysis (full model and sensitivity analyses) regarding outcome “SARS-CoV-2 seroconversion”.

|  | **Full model**^1^ | | **Sensitivity analysis 1** | | **Sensitivity analysis 3** | |
| --- | --- | --- | --- | --- | --- | --- |
|  |  |  | Treating cantons and  institutions as fixed effects | | persons without positively tested  household member | |
| Participants; Events | n=2’916; n=511 | | n=2’916: n=511 | | n=2’491: n=249 | |
| **Risk or protection factor** | **aOR (95% CI)** | **p** | **aOR (95% CI)** | **p** | **aOR (95% CI)** | **p** |
| **Sociodemographic data** |  |  |  |  |  |  |
| Age (per 10 years) | 0.96 (0.87 - 1.07) | 0.475 | 0.96 (0.86 - 1.06) | 0.422 | 0.92 (0.82 - 1.04) | 0.177 |
| Sex: male | 0.86 (0.62 - 1.18) | 0.349 | 0.86 (0.62 - 1.19) | 0.368 | 0.77 (0.53 - 1.13) | 0.176 |
| Living in Germany or Austria | 0.86 (0.47 - 1.58) | 0.628 | NA |  | 0.81 (0.42 - 1.55) | 0.522 |
| No of additional persons in households | 0.88 (0.79 - 0.98) | 0.016 | 0.87 (0.78 - 0.97) | 0.013 | 0.86 (0.76 - 0.98) | 0.022 |
| Child in household | 0.97 (0.71 - 1.34) | 0.859 | 0.98 (0.71 - 1.35) | 0.880 | 0.73 (0.48 - 1.10) | 0.132 |
| Any positively tested household member | 5.01 (3.89 - 6.46) | <0.001 | 5.11 (3.96 - 6.60) | <0.001 | NA |  |
| **Medical conditions** |  |  |  |  |  |  |
| Comorbidity | 0.93 (0.74 - 1.16) | 0.506 | 0.93 (0.74 - 1.16) | 0.507 | 0.96 (0.74 - 1.24) | 0.732 |
| Active smoking | 0.57 (0.41 - 0.79) | <0.001 | 0.57 (0.41 - 0.78) | 0.001 | 0.56 (0.39 - 0.82) | 0.002 |
| Pregnancy during study | 1.01 (0.56 - 1.82) | 0.979 | 0.99 (0.53 - 1.74) | 0.969 | 0.91 (0.45 - 1.86) | 0.802 |
| **Behaviour outside of work** |  |  |  |  |  |  |
| Prophylactic home remedies | 1.21 (0.90 - 1.63) | 0.205 | 1.21 (0.89 - 1.63) | 0.211 | 1.14 (0.81 - 1.62) | 0.448 |
| Social leisure activities | 1.02 (0.82 - 1.27) | 0.847 | 1.02 (0.82 - 1.27) | 0.863 | 0.92 (0.72 - 1.18) | 0.517 |
| Wearing a mask outside work | 0.96 (0.74 - 1.25) | 0.779 | 0.97 (0.74 - 1.27) | 0.833 | 0.92 (0.68 - 1.24) | 0.588 |
| Support for stronger public restrictions | 0.85 (0.64 - 1.14) | 0.282 | 0.88 (0.65 - 1.17) | 0.386 | 0.93 (0.67 - 1.29) | 0.657 |
| **HCW specifics** |  |  |  |  |  |  |
| Job: nurse | 1.55 (1.18 - 2.04) | 0.001 | 1.56 (1.19 - 2.06) | 0.002 | 1.83 (1.33 - 2.52) | <0.001 |
| Job: physician | 0.84 (0.59 - 1.21) | 0.360 | 0.84 (0.58 - 1.20) | 0.337 | 0.88 (0.57 - 1.36) | 0.562 |
| Full-time job (> 80%) | 1.08 (0.83 - 1.42) | 0.566 | 1.10 (0.84 - 1.44) | 0.509 | 1.11 (0.81 - 1.51) | 0.521 |
| Involved in AGP | 0.96 (0.75 - 1.24) | 0.775 | 0.95 (0.74 - 1.21) | 0.659 | 1.03 (0.78 - 1.37) | 0.820 |
| Working in ICU | 0.43 (0.27 - 0.68) | <0.001 | 0.43 (0.27 - 0.68) | <0.001 | 0.40 (0.22 - 0.71) | 0.002 |
| Frequency of contacts with COVID-19 patients | 1.51 (1.38 - 1.66) | <0.001 | 1.51 (1.38 - 1.67) | <0.001 | 1.50 (1.35 - 1.68) | <0.001 |
| Frequency of contacts with coworkers with COVID-19 | 1.22 (1.10 - 1.36) | <0.001 | 1.22 (1.10 - 1.36) | <0.001 | 1.18 (1.05 - 1.33) | 0.007 |
| **Behaviour at work** |  |  |  |  |  |  |
| Hygiene knowledge | 1.06 (0.77 - 1.46) | 0.698 | 1.03 (0.75 - 1.44) | 0.834 | 1.12 (0.76 - 1.65) | 0.562 |
| Regular meals in staff restaurant | 1.12 (0.88 - 1.41) | 0.363 | 1.12 (0.88 - 1.42) | 0.362 | 1.06 (0.81 - 1.40) | 0.657 |
| **Use of personal protective equipment** |  |  |  |  |  |  |
| Always used goggles^2^ | 0.91 (0.68 - 1.22) | 0.521 | 0.94 (0.70 - 1.26) | 0.677 | 0.99 (0.71 - 1.39) | 0.953 |
| Always used gloves/gown^2^ | 0.81 (0.59 - 1.11) | 0.182 | 0.80 (0.58 - 1.10) | 0.170 | 0.79 (0.55 - 1.13) | 0.197 |
| **Mostly used FFP2**^2^ | **0.73 (0.53 - 1.00)** | **0.053** | **0.76 (0.55 - 1.04)** | **0.088** | **0.69 (0.48 - 0.99)** | **0.046** |

^1^Institutions and living place (canton) included in model as random cluster term. For Sensitivity Analysis 2, the effects of these factors are not shown in the table.

^2^ In contact with COVID-19 patients outside of AGP

Abbreviations: HCW – Health care worker. AGP – Aerosol-generating procedure. FFP2 – Filtering facepiece class 2. NA – Not applicable.

Table S6. Subgroup analysis of HCW with frequent COVID-19 exposure *vs*. HCW with less frequent COVID-19 exposure; A) Cox regression (outcome SARS-CoV-2 positive swab); B) multivariable logistic regression (outcome SARS-CoV-2 seroconversion).

| **A) Cox Regression** |  |  |  |  |  |
| --- | --- | --- | --- | --- | --- |
|  | **1−20 COVID-19 patient contacts** | |  | **>20 COVID-19 patient contacts** | |
| Participants; Events | n = 1’292; n = 149 | |  | n = 1’120; n = 215 | |
| **Risk or protection factor** | **HR (95% CI)** | **p** |  | **HR (95% CI)** | **p** |
| **Exposure to COVID-19** |  |  |  |  |  |
| Patient exposure *(time dependent)* | 2.09 (1.66-2.62) | <0.001 |  | 1.52 (0.93-2.49) | 0.099 |
| Coworker exposure *(time dependent)* | 1.64 (1.09-2.46) | 0.018 |  | 1.61 (0.99-2.63) | 0.055 |
| Household exposure *(time dependent)* | 13.93 (9.64-20.12) | <0.001 |  | 6.49 (4.02-10.47) | <0.001 |
| Other exposure *(time dependent)* | 1.71 (1.36-2.14) | <0.001 |  | 1.20 (0.86-1.66) | 0.286 |
| Number of negative swabs *(time dependent)* | 0.88 (0.76-1.01) | 0.060 |  | 0.93 (0.83-1.03) | 0.143 |
| **Sociodemographic data** |  |  |  |  |  |
| Age (per 10 years) | 1.10 (0.99-1.22) | 0.086 |  | 1.05 (0.95-1.15) | 0.338 |
| Sex:male | 0.53 (0.29-0.98) | 0.042 |  | 0.86 (0.63-1.17) | 0.334 |
| Living in Germany or Austria | 1.20 (0.35-4.06) | 0.775 |  | 1.11 (0.49-2.52) | 0.794 |
| Child in household | 0.93 (0.65-1.34) | 0.710 |  | 1.24 (0.84-1.84) | 0.283 |
| **Medical conditions** |  |  |  |  |  |
| Comorbidity | 0.95 (0.71-1.27) | 0.705 |  | 0.99 (0.72-1.35) | 0.938 |
| Active smoking | 0.72 (0.38-1.36) | 0.307 |  | 0.61 (0.32-1.14) | 0.120 |
| Pregnancy during study | 1.41 (0.87-2.29) | 0.165 |  | 0.88 (0.40-1.94) | 0.745 |
| **Behaviour outside of work** |  |  |  |  |  |
| Prophylactic home remedies | 1.02 (0.70-1.49) | 0.909 |  | 1.09 (0.76-1.58) | 0.640 |
| Social leisure activities | 1.03 (0.76-1.41) | 0.836 |  | 0.91 (0.73-1.12) | 0.373 |
| Wearing a mask outside work | 0.70 (0.47-1.04) | 0.078 |  | 0.82 (0.61-1.11) | 0.193 |
| Support for stronger public restrictions | 0.91 (0.57-1.48) | 0.715 |  | 0.81 (0.65-1.02) | 0.076 |
| **HCW specifics** |  |  |  |  |  |
| Job: nurse | 1.20 (0.74-1.94) | 0.450 |  | 1.77 (1.30-2.41) | <0.001 |
| Job: physician | 0.53 (0.30-0.94) | 0.029 |  | 1.48 (0.91-2.39) | 0.111 |
| Full-time job (> 80%) | 1.10 (0.79-1.53) | 0.561 |  | 1.09 (0.85-1.39) | 0.504 |
| Involved in AGP | 1.08 (0.88-1.34) | 0.453 |  | 1.12 (0.76-1.65) | 0.566 |
| Working in intensive care | 0.64 (0.35-1.17) | 0.149 |  | 0.67 (0.45-0.99) | 0.046 |
| **Behaviour at work** |  |  |  |  |  |
| Hygiene knowledge | 1.12 (0.80-1.57) | 0.521 |  | 1.07 (0.78-1.46) | 0.691 |
| Regular meals in staff restaurant | 1.28 (0.89-1.84) | 0.186 |  | 1.02 (0.80-1.30) | 0.879 |
| **Use of personal protective equipment** |  |  |  |  |  |
| Always goggles^1^ | 0.76 (0.45-1.29) | 0.309 |  | 0.93 (0.68-1.27) | 0.664 |
| Always gloves/gown^1^ | 0.82 (0.42-1.61) | 0.562 |  | 1.28 (0.92-1.78) | 0.147 |
| **Mostly using FFP2 mask**^1^ | 1.06 (0.70-1.63) | 0.774 |  | 0.66 (0.54-0.81) | <0.001 |
|  |  |  |  |  |  |
| **B) Multivariable Logistic Regression** |  |  |  |  |  |
|  | **1−20 COVID-19 patient contacts** | |  | **> 20 COVID-19 patient contacts** | |
| Participants; Events | n = 1’156; n = 164 | |  | n = 1’019; n = 272 | |
| **Risk or protection factor** | **OR (95% CI)** | **p** |  | **OR (95% CI)** | **p** |
| **Exposure to COVID-19** |  |  |  |  |  |
| Frequency of contacts to coworkers with COVID-19 | 1.22 (1.02 - 1.45) | 0.032 |  | 1.23 (1.04 - 1.45) | 0.016 |
| Any positively tested household member | 3.97 (2.60 - 6.06) | <0.001 |  | 5.28 (3.59 - 7.78) | <0.001 |
| **Sociodemographic data** |  |  |  |  |  |
| Age (per 10 years) | 1.03 (0.86 - 1.22) | 0.776 |  | 0.91 (0.78 - 1.06) | 0.233 |
| Sex:male | 0.66 (0.37 - 1.20) | 0.175 |  | 0.95 (0.62 - 1.47) | 0.822 |
| Living in Germany or Austria | 1.28 (0.51 - 3.22) | 0.606 |  | 0.74 (0.35 - 1.57) | 0.427 |
| Number of additional persons in household | 0.94 (0.78 - 1.14) | 0.536 |  | 0.86 (0.74 - 1.01) | 0.066 |
| Child in household | 0.81 (0.46 - 1.41) | 0.450 |  | 1.08 (0.67 - 1.72) | 0.759 |
| **Medical conditions** |  |  |  |  |  |
| Comorbidity | 0.76 (0.52 - 1.13) | 0.173 |  | 1.02 (0.74 - 1.42) | 0.890 |
| Active smoking | 0.76 (0.46 - 1.27) | 0.296 |  | 0.49 (0.30 - 0.80) | 0.004 |
| Pregnancy during study | 1.13 (0.47 - 2.75) | 0.781 |  | 0.79 (0.28 - 2.24) | 0.657 |
| **Behaviour outside of work** |  |  |  |  |  |
| Prophylactic home remedies | 1.29 (0.80 - 2.10) | 0.298 |  | 1.11 (0.69 - 1.80) | 0.665 |
| Social leisure activities | 1.02 (0.71 - 1.46) | 0.931 |  | 1.10 (0.80 - 1.52) | 0.545 |
| Wearing a mask outside work | 0.82 (0.51 - 1.29) | 0.388 |  | 1.18 (0.80 - 1.74) | 0.404 |
| Support for stronger public restrictions | 1.01 (0.62 - 1.66) | 0.970 |  | 0.83 (0.54 - 1.27) | 0.395 |
| **HCW specifics** |  |  |  |  |  |
| Job: nurse | 1.39 (0.89 - 2.17) | 0.151 |  | 2.22 (1.40 - 3.52) | <0.001 |
| Job: physician | 0.50 (0.26 - 0.96) | 0.038 |  | 1.33 (0.78 - 2.28) | 0.298 |
| Full-time job (> 80%) | 1.31 (0.82 - 2.08) | 0.254 |  | 0.97 (0.64 - 1.48) | 0.901 |
| Involved in AGP | 1.35 (0.90 - 2.03) | 0.144 |  | 0.82 (0.58 - 1.15) | 0.251 |
| Working in intensive care | 0.33 (0.11 - 1.00) | 0.049 |  | 0.51 (0.30 - 0.87) | 0.015 |
| **Behaviour at work** |  |  |  |  |  |
| Hygiene knowledge | 0.99 (0.59 - 1.65) | 0.968 |  | 1.12 (0.65 - 1.93) | 0.691 |
| Regular meals in staff restaurant | 1.53 (1.03 - 2.28) | 0.037 |  | 1.03 (0.72 - 1.47) | 0.886 |
| **Use of personal protective equipment** |  |  |  |  |  |
| Always goggles^1^ | 0.90 (0.54 - 1.51) | 0.703 |  | 1.06 (0.72 - 1.58) | 0.755 |
| Always gloves/gown^1^ | 0.60 (0.35 - 1.03) | 0.065 |  | 0.98 (0.64 - 1.49) | 0.915 |
| **Mostly using FFP2 mask**^1^ | **0.75 (0.42 - 1.32)** | **0.320** |  | **0.64 (0.42 - 0.97)** | **0.036** |

^1^ In contact with COVID-19 patients outside of AGP

Abbreviations: HCW – Health care worker. AGP – Aerosol-generating procedure. FFP2 – Filtering facepiece class 2. NA – not applicable.

Table S7. Subgroup analysis of HCW performing AGP: A) Cox regression (outcome SARS-CoV-2 positive swab); B) multivariable logistic regression (outcome SARS-CoV-2 seroconversion).

| **A) Cox Regression** |  |  |
| --- | --- | --- |
|  | **Participants involved in AGP** | |
| Participants; Events | n = 1’204; n = 188 | |
| **Risk or protection factor** | **HR (95% CI)** | **p** |
| **Exposure to COVID-19** |  |  |
| Patient exposure *(time dependent)* | 1.50 (0.98-2.32) | 0.065 |
| Coworker exposure *(time dependent)* | 1.72 (1.24-2.37) | 0.001 |
| Household exposure *(time dependent)* | 9.75 (6.28-15.14) | <0.001 |
| Other exposure *(time dependent)* | 1.42 (1.07-1.88) | 0.015 |
| Number of negative swabs *(time dependent)* | 0.88 (0.75-1.03) | 0.108 |
| **Sociodemographic data** |  |  |
| Age (per 10 years) | 1.02 (0.88-1.19) | 0.776 |
| Sex:male | 0.87 (0.57-1.33) | 0.531 |
| Living in Germany or Austria | 1.46 (0.79-2.67) | 0.224 |
| Child in household | 1.15 (0.88-1.50) | 0.297 |
| **Medical conditions** |  |  |
| Comorbidity | 1.13 (0.85-1.49) | 0.402 |
| Active smoking | 0.65 (0.37-1.13) | 0.129 |
| Pregnancy during study | 0.56 (0.13-2.38) | 0.433 |
| **Behaviour outside of work** |  |  |
| Prophylactic home remedies | 1.11 (0.67-1.83) | 0.685 |
| Social leisure activities | 0.98 (0.77-1.26) | 0.900 |
| Wearing a mask outside work | 0.75 (0.51-1.11) | 0.157 |
| Support for stronger public restrictions | 0.91 (0.69-1.20) | 0.512 |
| **HCW specifics** |  |  |
| Job: nurse | 1.17 (0.87-1.58) | 0.309 |
| Job: physician | 1.12 (0.64-1.96) | 0.697 |
| Full-time job (> 80%) | 0.95 (0.65-1.37) | 0.767 |
| Working in intensive care | 0.77 (0.48-1.24) | 0.277 |
| **Behaviour at work** |  |  |
| Hygiene knowledge | 1.72 (1.03-2.88) | 0.039 |
| Regular meals in staff restaurant | 1.00 (0.67-1.47) | 0.981 |
| **Use of personal protective equipment** |  |  |
| Always goggles^1^ | 0.78 (0.59-1.03) | 0.075 |
| Always gloves/gown^1^ | 1.11 (0.67-1.82) | 0.686 |
| **Mostly using FFP2 mask^1^** | **0.71 (0.50-1.01)** | **0.059** |
| Always using FFP2 mask during AGP | 1.08 (0.71-1.64) | 0.726 |
|  |  |  |
| **B) Multivariable Logistic Regression** |  |  |
|  | **Participants involved in AGP** | |
| Participants; Events | n = 1’087; n = 216 | |
| **Risk or protection factor** | **OR (95% CI)** | **p** |
| **Exposure to COVID-19** |  |  |
| Frequency of contacts to COVID-19 patients | 1.44 (1.22 - 1.70) | <0.001 |
| Frequency of contacts to coworkers with COVID-19 | 1.27 (1.06 - 1.51) | 0.008 |
| Any positively tested household member | 4.69 (3.14 - 7.00) | <0.001 |
| **Sociodemographic data** |  |  |
| Age (per 10 years) | 1.01 (0.85 - 1.19) | 0.951 |
| Sex:male | 0.99 (0.64 - 1.53) | 0.953 |
| Living in Germany or Austria | 1.38 (0.65 - 2.91) | 0.404 |
| Number of additional persons in households | 0.89 (0.75 - 1.06) | 0.185 |
| Child in household | 0.90 (0.53 - 1.52) | 0.693 |
| **Medical conditions** |  |  |
| Comorbidity | 1.08 (0.76 - 1.52) | 0.667 |
| Active smoking | 0.46 (0.28 - 0.78) | 0.004 |
| Pregnancy during study | 1.25 (0.39 - 3.95) | 0.708 |
| **Behaviour outside of work** |  |  |
| Prophylactic home remedies | 1.41 (0.88 - 2.25) | 0.149 |
| Social leisure activities | 1.05 (0.75 - 1.47) | 0.786 |
| Wearing a mask outside work | 0.84 (0.56 - 1.26) | 0.404 |
| Support for stronger public restrictions | 1.11 (0.73 - 1.69) | 0.633 |
| **HCW specifics** |  |  |
| Job: nurse | 1.28 (0.78 - 2.09) | 0.330 |
| Job: physician | 0.97 (0.55 - 1.73) | 0.930 |
| Full-time job (> 80%) | 0.95 (0.62 - 1.46) | 0.822 |
| Working in intensive care | 0.44 (0.27 - 0.74) | 0.002 |
| **Behaviour at work** |  |  |
| Hygiene knowledge | 1.81 (0.94 - 3.50) | 0.077 |
| Regular meals in staff restaurant | 1.09 (0.75 - 1.59) | 0.645 |
| **Use of personal protective equipment** |  |  |
| Always used goggles^1^ | 0.89 (0.59 - 1.32) | 0.552 |
| Always used gloves/gown^1^ | 0.86 (0.56 - 1.34) | 0.518 |
| **Mostly used FFP2 mask**^1^ | **0.66 (0.43 - 1.01)** | **0.055** |
| Always used FFP2 mask during AGP | 0.89 (0.62 - 1.28) | 0.535 |

^1^ In contact with COVID-19 patients outside of AGP

Abbreviations: HCW – Health care worker. AGP – Aerosol-generating procedure. FFP2 – Filtering facepiece class 2. NA – not applicable.
